# A computational lens on menopause-associated psychosis

**DOI:** 10.3389/fpsyt.2022.906796

**Published:** 2022-08-03

**Authors:** Victoria L. Fisher, Liara S. Ortiz, Albert R. Powers

**Affiliations:** Yale University School of Medicine and the Connecticut Mental Health Center, New Haven, CT, United States

**Keywords:** menopause, psychosis, computational psychiatry, predictive coding, estrogen

## Abstract

Psychotic episodes are debilitating disease states that can cause extreme distress and impair functioning. There are sex differences that drive the onset of these episodes. One difference is that, in addition to a risk period in adolescence and early adulthood, women approaching the menopause transition experience a second period of risk for new-onset psychosis. One leading hypothesis explaining this menopause-associated psychosis (MAP) is that estrogen decline in menopause removes a protective factor against processes that contribute to psychotic symptoms. However, the neural mechanisms connecting estrogen decline to these symptoms are still not well understood. Using the tools of computational psychiatry, links have been proposed between symptom presentation and potential algorithmic and biological correlates. These models connect changes in signaling with symptom formation by evaluating changes in information processing that are not easily observable (latent states). In this manuscript, we contextualize the observed effects of estrogen (decline) on neural pathways implicated in psychosis. We then propose how estrogen could drive changes in latent states giving rise to cognitive and psychotic symptoms associated with psychosis. Using computational frameworks to inform research in MAP may provide a systematic method for identifying patient-specific pathways driving symptoms and simultaneously refine models describing the pathogenesis of psychosis across all age groups.

## Introduction

Schizophrenia is a debilitating disorder associated with adverse social, psychological, and biological effects. Most individuals experience their first psychotic episode in their third or fourth decade of life 20–30s (1, 2). During early adulthood, men are 40% more likely to experience their first episode of psychosis (3, 4). However, as women approach menopause, there is an uptick in first-episode psychosis and hospital admissions not seen in men of the same age (5, 6). This menopause-associated psychosis (MAP) is widely recognized; however, very little work has focused on the underlying mechanisms that connect the menopausal transition to psychosis.

It has been proposed that the sudden decline in reproductive hormones during the menopause transition may trigger MAP. The Estrogen Protective Hypothesis posits that estrogen protects against psychotic symptom emergence (7). Evidence for this hypothesis comes from research demonstrating an inverse relationship between estrogen levels and psychotic symptoms: women with schizophrenia have lower estrogen levels (8); hospital admissions for psychosis increase during periods associated with low estrogen [luteal phase of menstruation, menopause, and post-partum (9–15)]; further, there is evidence, albeit less consistent, that estrogen treatments and contraceptives supplement antipsychotics in reducing symptoms (16–18). While there is evidence linking low estrogen levels with psychosis, there has been limited focus on how estrogen may alter the underlying processes that contribute to psychotic states.

**FIGURE 1:**
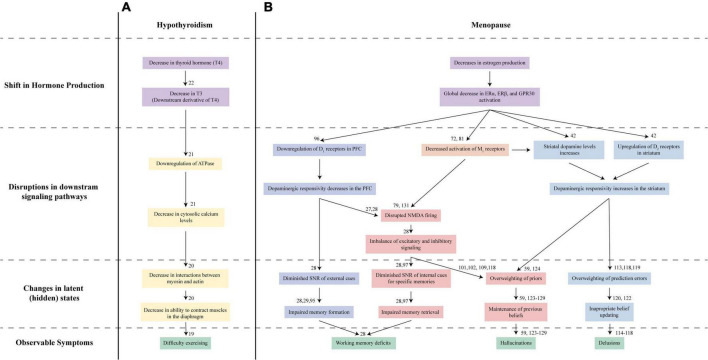
Proposed Nosology of Estrogen-Mediated Psychosis. Understanding hypothyroidism-mediated exercise difficulties **(A)** and MAP **(B)** requires looking at multiple levels of changes. **(A)** In hypothyroidism, the initial decrease in thyroid hormone T4 (purple) and its derivative T3 leads to a series of disruptions in the downstream signaling pathways (yellow). These disruptions ultimately lead to impaired global muscle contractions in the diaphragm resulting in symptoms like irregular breathing and difficulty exercising (green). **(B)** Our proposed mechanism for MAP reflects this multilevel approach to understanding disorders. Diminished estrogen (purple) signaling in the brain affects bottom-up (blue) and top-down (red/orange) mechanisms that contribute to the latent states driving working memory issues, hallucinations, and delusions (green) characteristic of psychosis. Estrogen decline may disrupt signaling across several pathways: dopaminergic D_1_ receptor activation in the prefrontal cortex (dark blue), D_2_ receptor activation in the striatum (light blue), cholinergic M_1_ activation (orange), and glutamatergic NMDA receptor activation (red). Disruptions to these symptoms, in turn, lead to altered latent states, driving psychosis development.

Understanding MAP requires the same multifaceted approach as other complex medical disorders, which have signs and symptoms that arise as the result of underlying pathophysiological processes that are not directly observable. Take, for instance, hypothyroidism. Patients with this disorder may first present with symptoms like difficulty with exercising and breathing issues (19). These observable symptoms arise as the result of processes that are only observed on specific testing: a cascade of abnormalities starting with low thyroid hormone T4 and progressing to decreased T3 levels, downregulation of ATPase, low cytosolic calcium levels, and finally, effects on muscle tissues leading to weakness and the difficulty breathing that lead to presenting symptoms (Figure 1A) (19–22).

Placing the action of estrogen and other reproductive hormones within a computational framework may help elucidate how the menopausal transition leads to positive and cognitive symptoms (Figure 1B). Computational psychiatry has been instrumental to uncovering the links between biological mechanisms and behavioral abnormalities observed in psychiatric disorders (23–25) *via* the identification of latent (unobserved) states driving behavior and instantiated by specific neural circuits (26–29). This feature is a major advantage of the approach, allowing for the generation of falsifiable hypotheses about symptom development and driving iterative refinement of models of psychiatric disease. As applied to our present question, computational models facilitate hypothesis generation about biological mechanisms that may drive latent states linked to psychotic symptom development during the menopause transition.

In this perspective, we aim to critically evaluate the current literature on estrogen and psychosis and how computational frameworks may help to explain psychosis onset in light of modulations in estrogen. We begin with a discussion of how estrogen signaling in the brain influences neurotransmitter signaling abnormalities present in schizophrenia. We then discuss how these systems correspond to elements of computational frameworks accounting for positive and cognitive symptoms that precede and define psychosis. Lastly, we propose future work testing hypotheses directly arising from our framework.

## Estrogen and neurotransmitter systems

### Estrogen signaling in the brain

Historically, it was thought that estrogen predominantly affected gene expression; however, research has highlighted estrogen has far more diverse effects on a myriad of neurotransmitter systems and functions (30–32). Estrogen interacts with the central nervous system through three primary receptors: ERα (33), ERβ (34, 35), and GPR30 (36). Of particular relevance to schizophrenia is estrogen activity at ERα and ERβ, which are located in the prefrontal cortex (PFC), dorsal striatum, nucleus accumbens, and hippocampus (30). Disrupted signaling in these areas is implicated in cognitive and positive symptoms that characterize psychosis (37–39). In this section, we will explore how modulation by estrogen affects neurotransmitter signaling associated with psychosis.

### Dopamine

The dopamine hypothesis posits that positive symptoms of schizophrenia are associated with hyperactive dopaminergic signaling, specifically D_2_-receptor networks in the striatum. Estrogen receptors are also highly expressed in the striatum (30); however, the role of estrogen in regulating dopamine levels is not clear. Evidence suggests that estrogen increases levels of striatal dopamine in female rats (40) likely through the inhibition of dopamine reuptake proteins (41). Conversely, ovariectomized (i.e., estrogen-free) rats display downregulated expression of dopamine reuptake proteins and increased D_2_ receptors in the striatum and nucleus accumbens (42), indicating that estrogen would typically result in lower dopamine levels. Typical receptor expression is restored after treatment with 17β-estradiol (42).

The dopamine hypothesis additionally posits that diminished activation of D_1_ receptors in the PFC contributes to the cognitive deficits commonly seen in psychotic spectrum disorders (43). Cognitive symptoms are the most significant predictors of disease prognosis in schizophrenia (44). Estrogen has a strong preventative effect on cognitive decline, substantially affecting verbal memory (45, 46). Estrogen therapy has even been found to diminish cognitive decline associated with both aging and schizophrenia (47–49) [but see (50–53) for conflicting data]. Importantly, estrogen’s effects on cognition also appear to be dopamine-dependent as studies in women have shown that increases in estrogen (either by natural fluctuation or treatments) predominantly help women with inherently low levels of dopamine (54, 55).

### Acetylcholine

Acetylcholine has also been identified as a potential modulator of cognitive and psychotic symptoms. Acetylcholine targets two primary receptors families: muscarinic (mAChRs) (56) and nicotinic (nAChRs) (57). Individuals with schizophrenia display altered muscarinic and nicotinic signaling (58). However, our discussion of acetylcholine and its relation to MAP will be limited to mAChRs, as muscarinic receptor modulation is more predominantly featured in computational models (59, 60), and mAChRs are targets of new antipsychotics (61, 62).

Positive symptoms are associated with reduced activation of mAChRs. Reduced muscarinic activity induces a psychosis-like state and may worsen pre-existing symptoms in schizophrenia (58). The relationship between mAChR and positive symptoms has explicitly been linked with a reduction in M_1_ and M_4_ receptors. Post-mortem studies in individuals with schizophrenia demonstrate reduced M_1_ and M_4_ receptor density in the hippocampus and striatum (58, 63). M_1_ and M_4_ agonists have been shown to reduce positive and negative symptoms in schizophrenia (63–66). Rodent models suggest this relationship may be partly attributed to the inverse correlation between M_1_ receptor density and striatal dopamine levels (67) and that M_4_ receptor activation mediates dopaminergic release in the striatum (65, 68).

Estrogen’s protection against positive symptoms may be partially due to its influence on muscarinic receptor expression and activation. Estrogen has been shown to enhance acetylcholine release and decrease uptake in rodents (69, 70) and women (71). Women with surgically induced menopause have lower global M_1_ and M_4_ receptor density (72). Estradiol treatments increased global expression of these receptors with significant increases observed in the thalamus, lateral frontal cortex, and notably the hippocampus and left striatum (72). However, these effects run contrary to those seen in rodent models after ovariectomy, after which either down-regulation or no effect on mAChRs in the hippocampus was observed (73–75). While differences may be attributed to the timing of estrogen treatment and age at ovariectomy, more research is needed to clarify the exact relationship between estrogen and acetylcholine.

Estrogen may also protect against cognitive deficits through cholinergic pathways (76). Reduced mAChR activation disrupts memory functioning and attention, and substantial disruption can even shut down cognitive processes entirely (77). The influence of acetylcholine on cognition may be mediated by its modulatory effect on glutamatergic NMDA receptor activation. Reduced activation of NMDA receptors in the hippocampus is associated with cognitive impairment (39, 78). Rodent models suggest that muscarinic receptors colocalize with NMDA and increase the potentiation of NMDA networks (79).

Estrogen influences cognition through muscarinic receptors. Ovariectomized rats display diminished cognitive functioning, which is accompanied by disruptions in typical acetylcholine synthesis and reuptake (80, 81). However, these cognitive deficits and disruptions in acetylcholine maintenance can be improved after estrogen treatments (82). Estrogen has been found to improve cognition after inhibition of M_1_ receptors (83–88). Mechanisms for the effect of estrogen on cognition may be that ERα promotes neuronal growth and acetylcholine synthesis in the basal forebrain, as seen in mice (89). Rodent models also demonstrate that estrogen improves NMDA potentiation in the hippocampus and overcomes cognitive deficits induced by NMDA antagonists (90).

## Computational frameworks

Estrogen affects dopaminergic, cholinergic, and glutamatergic neural signaling implicated in schizophrenia. However, the overlap in these pathways is not sufficient to determine how estrogen loss during menopause may induce MAP. Understanding the mechanisms by which estrogen deficits may cause cognitive and positive symptoms may illustrate how estrogen decline leads to psychosis. Thus, to address this gap, we will explore how a sudden decline in estrogen may affect the neural processes implicated in the generation of cognitive and positive symptoms in schizophrenia. These are also summarized in Figure 1.

### Cognition

Cognitive impairments are pervasive in schizophrenia, with measures of cognitive functioning as one of the strongest predictors of psychosis conversion and everyday functioning. In particular, working memory is substantially impaired across phases of illness, from those at high risk of conversion to those with chronic schizophrenia (91). Estrogen has also been shown to improve working memory in post-menopausal women (92). Indicating that psychosis development during menopause may be explained through underlying latent states that drive working memory deficits. Research in computational psychiatry has identified diminished signal-to-noise ratio (SNR) as a primary contributor to working memory deficits (28). We will explore how estrogen decline may impact SNR, which may contribute to working memory deficits in MAP.

Two computational models that formalize the relationship between SNR aberrations and working memory deficits are connectionist and attractor network models. Connectionist models use networks of computational units (representing neurons) to form artificial neural networks (93). Aberrations in neural processing can be introduced to artificial networks by altering the properties of these units. These alterations can mimic abnormalities observed biologically, such as responsiveness to neurotransmitter signals (gain) (28, 93, 94). Studies using *in silico* connectionist models have found that reducing gain from dopaminergic signaling in the PFC (i.e., lowering SNR) led to cognitive deficits akin to those observed in schizophrenia (28) and decreased maintenance of important contextual information, often associated with working memory deficits (28, 29, 95).

Estrogen modulation of D_1_ receptor activation in the PFC may increase SNR, preventing working memory decline and, subsequently, psychosis. Jacobs and D’Esposito found that estrogen increased dopaminergic signaling in the PFC when needed for cognitive tasks (54). However, this effect was only helpful in women who had a genetic propensity toward low dopamine levels, indicating that estrogen is important in normalizing dopaminergic signaling specifically in hypodopaminergic states. In addition to direct action at dopamine receptors, estrogen may upregulate dopamine receptor expression. Research in ovariectomized rats found that stimulating ERβ increased D_1_ receptor expression in the PFC (96). Thus, estrogen decline during the menopause transition may diminish SNR *via* decreased PFC responsivity to dopamine, spurring working memory deficits that precede psychosis. However, further research is needed to confirm this pathway toward illness.

Evidence from attractor network models is consistent with findings from connectionist models. Attractor networks are a group of neurons that form a stable firing pattern due to excitatory modulation within the network (97). Attractor networks are composed of neural units designed to reflect memory formation and retrieval using attractor models (98, 99). Additionally, low SNR can be introduced to these computational models by decreasing the probability of excitatory (NMDA) neurons firing, which limits the memory retrieval process (28, 100). Research in attractor networks found that D_1_ stimulation modulated both excitatory NMDA and inhibitory GABA signaling within attractor networks, increasing SNR and improving memory retrieval (27, 28), replicating findings using NMDA antagonists (101, 102). Thus, illustrating that modulation of dopaminergic, glutamatergic, and gabaergic signaling may disrupt working memory leading individuals to a pre-psychotic state.

Estrogen decline during menopause may disrupt excitatory signaling at NMDA receptors needed for memory retrieval, leading to working memory issues. As explored previously, a decrease in estrogen may impair working memory by decreasing the SNR of D_1_-modulated neural networks in the prefrontal cortex to dopamine, which in turn destabilizes NMDA and GABA firing (28). Another potential route by which estrogen affects working memory is through cholinergic modulation of NMDA receptors. Estrogen has been shown to increase NMDA receptor binding in the hippocampus (103). This may be due to its ability to counteract inhibition of M_1_ receptors (81), which co-localize with NMDA receptors and induce NMDA firing (79). Estrogen’s effect on the M_1_-NMDA/GABA network mimics the actions of newly developed antipsychotics, which aim to improve cognition by increasing activation of M_1_ receptors (104, 105). This illustrates that if a system is reliant on estrogen to maintain functionality within this network, a sudden decline in estrogen may lead to diminished excitatory signals and disruptions in working memory (28).

### Positive symptoms

Diminished estrogen signaling in the brain may contribute to the development of positive symptoms, such as hallucinations and delusions. The mechanisms underlying hallucination and delusion formation have been explored extensively through the lens of predictive coding theory. In this Bayesian framework, individuals build and update an internal model of the world using incoming sensory evidence (106–108). Within schizophrenia, disruptions in both model updating and model-based inference are associated with positive symptoms (60, 109).

In predictive coding, internal models are constantly being altered *via* belief updating to account for changes in the world. Belief updating occurs due to a discrepancy between the expectation based on the internal model (prediction) and the incoming sensory information, termed a prediction error (PE). Not all PEs contribute equally to model updating, which is driven not only by the magnitude of PEs, but the weight (or precision) they are afforded (110–112). However, there is evidence that mechanisms for appropriate weighting of PEs are disrupted in schizophrenia (113). This disruption leads to inappropriate belief updating, which may result in delusions and hallucinations (114–118).

Aberrations in dopaminergic signaling in the striatum contribute to delusions and hallucinations. Dopamine is believed to increase the weighting of reward prediction errors (113, 118, 119): D_2_ signaling in the striatum strengthens associations between stimuli that reliably predict reward (120, 121). Similarly, hyperdopaminergic signaling in the striatum may lead to increased precision of PEs, promoting inappropriate belief formation (120, 122). Fittingly, overly precise PEs have been tied to increased delusional ideation (60, 116).

Estrogen removal may increase D_2_ receptor activation leading to inappropriate model updating and delusions. Neural modulations causing sudden increases in D_2_ receptor activation may induce psychotic states (60, 121). Ovariectomized rats demonstrate estrogen modulated increases in D_2_ receptor expression and dopamine production (42). A sudden increase in D_2_ receptors would amplify dopaminergic signaling and increase the precision of PE, shifting internal models toward inappropriate new belief formation. Estrogen deficits may also increase striatal dopamine levels due to diminished acetylcholine signaling, which may be attributed to a reduction in M_1_ receptors, also observed after ovariectomy (72). However, given conflicting evidence surrounding if estrogen leads to increases or decreases in dopamine production, more research is needed to clarify the exact mechanism by which estrogen decline affects dopaminergic signaling in the striatum.

Hallucination development has also been formulated in light of the predictive processing theory. Hallucinations are thought to arise due to an overweighting of priors relative to incoming sensory evidence (59, 123–129). The processes leading to hallucinations (i.e., overweighting priors) and delusions (i.e., overweighting sensory evidence) may seem contradictory. This seeming contradiction is often resolved through appeals to hierarchy: delusions may represent aberrations at lower level processing, while hallucinations reflect aberrations at higher levels (118). Separation across the processing hierarchy may also be paired with separation over time: PE-mediated belief formation may itself lead to the solidification of inappropriate beliefs and subsequent hallucination formation, at least in a subgroup of individuals with psychosis (130).

Lastly, while there is evidence that dopamine signaling at D_2_ receptors leads to higher prior precision (59, 124), diminished excitatory (NMDA) signaling may be responsible for disruptions at higher levels of processing (101, 102, 109, 118). Disrupted signaling at NMDA receptors due to menopause may underscore hallucination formation. A sharp decline in M_1_ receptor activation can disrupt signaling at NMDA receptors (131). While the M_1_-modulated effects of estrogen on NMDA activation are more closely associated with cognition, these effects may also promote overweighting of prior beliefs.

Taken together, evidence suggests estrogen impacts symptom expression *via* multiple neurotransmitter systems. While efforts have been made to form a unifying theory underlying psychosis (118), future work should aim to link estrogen levels, neurotransmitter signaling, belief formation and updating, and phenomenology of positive psychotic symptoms. We discuss possible routes toward this future work below.

## Discussion and guidance for future directions

In this perspective manuscript, we used computational and biochemical models of cognitive and psychotic symptoms to generate hypotheses about how menopause triggers psychosis. Understanding what biological changes lead to changes in observable behaviors is essential for early intervention and treatment. Identifying risk factors for psychosis has been imperative for the prevention and early treatment of traditionally recognized psychosis. Thus, the next step is to use these computational models to pinpoint what causes psychosis in women during the menopausal transition.

We can best understand the MAP transition by replicating work done on the period leading up to schizophrenia. The most influential research to characterize this period and predict psychosis onset comes from large longitudinal studies (132–136). However, the current studies did not focus on factors that may underlie MAP, such as a decline in reproductive hormones. To fill this gap, studies should track hormonal fluctuations and changes in psychotic symptoms in women as they go through menopause. Despite the insights that may be gained from this line of research, it provides a limited understanding of what elements are driving psychosis, particularly due to the substantial variability in measured hormone levels both within and between women. These limitations necessitate computational models of psychosis that incorporate biological, behavioral, and symptom changes to test hypotheses of the underlying neural mechanisms and latent states driving MAP.

Computational models provide immense benefits due to their capacity to disentangle underlying mechanisms of psychosis. Understanding these mechanisms may guide specific interventions for women going through menopause or even facilitate patient-specific treatments. Computational research on estrogen and MAP has broad implications as well. It may illustrate how estrogen contributes to psychotic episodes in individuals with illnesses associated with hormonal dysregulation (137–139). Additionally, it may illuminate the mechanisms underlying general differences between men and women with schizophrenia (140). Further, computational methods may highlight specific aspects of psychosis that estrogen alone cannot explain, thereby facilitating targeted research into how other hormones, such as androgens (141, 142) and neurosteroids (143), affect psychosis. This proposed multifaceted approach may be crucial to improving our understanding of psychosis and orienting future research.

## Data availability statement

The original contributions presented in the study are included in the article/supplementary material, further inquiries can be directed to the corresponding author/s.

## Author contributions

VF wrote the first draft of the main manuscript. AP provided edits and guidance on the conceptual framework. LO provided edits and guidance on Hypothyroidism. All authors contributed to the article and approved the submitted version.
